# Combined Low Plant and High Animal Dietary Pattern Is Associated with a Lower Risk of Anemia among Dyslipidemic Adult Women in Taiwan: A Retrospective Study from 2001 to 2015

**DOI:** 10.3390/ijerph19106231

**Published:** 2022-05-20

**Authors:** Sintha Dewi Purnamasari, Chien-Yeh Hsu, Yi-Tien Chen, Adi Lukas Kurniawan, Hsiu-An Lee, Jane C.-J. Chao

**Affiliations:** 1 School of Nutrition and Health Sciences, College of Nutrition, Taipei Medical University, 250 Wu-Hsing Street, Taipei 110, Taiwan; sinthadewips@almaata.ac.id (S.D.P.); 8lukas@ntunhs.edu.tw (A.L.K.); 2 Department of Nutrition, Faculty of Health Science, Universitas Alma Ata, Yogyakarta 55183, Indonesia; 3 Alma Ata Center for Healthy Life and Foods (ACHEAF), Universitas Alma Ata, Yogyakarta 55183, Indonesia; 4 Department of Information Management, National Taipei University of Nursing and Health Sciences, 365 Ming-Te Road, Peitou District, Taipei 112, Taiwan; cyhsu@ntunhs.edu.tw; 5 Master Program in Global Health and Development, College of Public Health, Taipei Medical University, 250 Wu-Hsing Street, Taipei 110, Taiwan; 6 School of Food Safety, College of Nutrition, Taipei Medical University, 250 Wu-Hsing Street, Taipei 110, Taiwan; ytc@tmu.edu.tw; 7 Research Center for Healthcare Industry Innovation, National Taipei University of Nursing and Health Sciences, 365 Ming-Te Road, Peitou District, Taipei 112, Taiwan; 8 National Health Research Institutes, 35 Keyan Road, Zhunan Town, Miaoli County 350, Taiwan; billy72325@gmail.com; 9 Nutrition Research Center, Taipei Medical University Hospital, 252 Wu-Hsing Street, Taipei 110, Taiwan

**Keywords:** dyslipidemia, anemia, plant diet, animal diet

## Abstract

Anemia and dyslipidemia often occurs in premenopausal women. This study investigated the association between dietary patterns and anemia among dyslipidemic women in Taiwan. This study recruited 22,631 dyslipidemic women aged 20–45 years between 2001 and 2015. The dietary assessment was collected by a validated food frequency questionnaire. The biochemical data including blood lipids, red blood cells, hemoglobin, hematocrit, and C-reactive protein (CRP) were retrieved from the database. Women with a combined high plant diet (HP) and low animal diet (LA) were associated with a lower prevalence of obesity (11.7%), central obesity (16.0%), high total cholesterol (16.4%), and high low-density lipoprotein cholesterol (11.9%), and had lower hemoglobin (12.9 ± 1.4 g/dL), hematocrit (38.8 ± 3.6%), and CRP levels (20.6 ± 31.4 nmol/L). The low plant diet (LP) + high animal diet (HA) pattern was negatively associated with moderate to severe anemia (OR: 0.76, 95% CI: 0.64–0.92, *p* = 0.004) compared to the low plant diet (LP) + low animal diet (LA) pattern. However, the HP + LA pattern was positively correlated with moderate to severe anemia (OR: 1.22, 95% CI: 1.04–1.43, *p* = 0.015). In conclusion, a low plant and high animal diet plays a role in preventing anemia development among dyslipidemic women.

## 1. Introduction

The prevalence of dyslipidemia in Taiwan has been increasing because of changes in lifestyle and eating habits. According to the findings of the Nutrition and Health Survey in Taiwan (NAHSIT) 2016–2019, the prevalence of hyperlipidemia, defined as triglycerides (TG) ≥ 2.26 mmol/L, total cholesterol (TC) ≥ 6.21 mmol/L, or antihyperlipidemic medication in women aged 20–29, 30–39, and 40–49 years, were 5.1%, 8.0%, and 14.0%, respectively [1]. Hyperlipidemia, a type of dyslipidemia, was characterized by abnormally elevated lipid levels [2]. Dyslipidemia is defined as an abnormal lipid profile that includes elevated blood lipids in triglycerides (TG), total cholesterol (TC), low-density lipoprotein cholesterol (LDL-C), or low high-density lipoprotein cholesterol (HDL-C) [3]. The previous study found that the vegetables-fruits-seafood dietary pattern was correlated with reducing the risk of hypercholestrolemia [4]. However, high intakes of animal foods such as red meat, eggs, milk, and dairy products have been reported to be correlated with higher risks for hypercholesterolemia and cardiovascular disease [5]. Recent evidence suggests that the popularity of plant-based food was increasing among teenagers and young people, especially women [6]. Premenopausal women seem to be a high-risk group of people who are often diagnosed with anemia, especially in regard to iron loss experienced during menstruation and pregnancy [7]. There is still a debate whether vegetarians are able to have the best quantities of essential nutrients, such as vitamins B_12_ and D, iron, calcium, and n-3 long-chain fatty acids. In addition, heme iron can be more easily absorbed in terms of bioavailability than nonheme iron. Thus, anemia is one of the most concerning diseases for vegetarians [8]. Additionally, diet is not a simple sum of several nutrients, as each individual food contains a mixture of nutrients associated with multiple compounds of limited or unknown nutritional value [9]. Considering the increasing prevalence of dyslipidemia, vulnerable to anemia, and an unbalanced dietary intake in premenopausal women in Taiwan, our motivation and novelty was to study whether dietary patterns based on plant or animal sources were correlated with the risk of anemia in adult women with dyslipidemia in Taiwan using a 15-year database. We hypothesized that the dietary pattern could play a role in the development of anemia among dyslipidemic women in Taiwan. Therefore, our study aimed to investigate the association of low or high plant/animal dietary patterns with anemia related biomarkers such as red blood cells, hemoglobin, and hematocrit, and the severity of anemia among dyslipidemic women.

## 2. Materials and Methods

### 2.1. Data Source and Study Subjects

**Figure 1:**
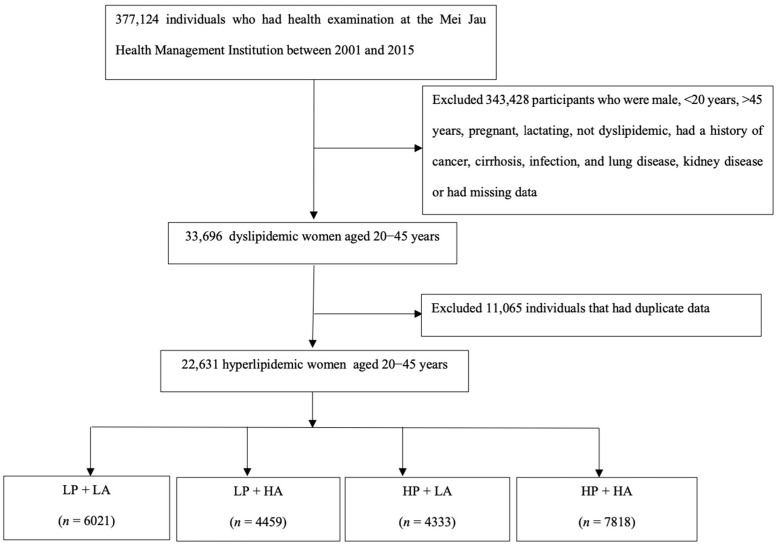
Flow chart diagram of subjects included in the study. LP: low plant diet, LA: low animal diet, HA: high animal diet, HP: high plant diet.

This retrospective study used the secondary data between 2001 and 2015 from the Mei Jau Health Management Institute database. The Mei Jau Health Management Institute provided physical examinations at four health screening centers in Taipei, Taoyuan, Taichung, and Kaohsiung, and collected population-based health data on a longitudinal scale [10]. Before taking the physical examination, all the participants spent 20–30 min for completing a self-reported questionnaire to obtain information on sociodemographic data, lifestyle, medical history, and dietary habits [11]. A series of medical tests including blood tests, anthropometric measurements, and physical examination for all the participants were then conducted by the medical professionals at the Mei Jau health screening center on the same day when they had an appointment for physical examination. The study was approved by the Taipei Medical University-Joint Institutional Review Board (no. 201907024). All participants signed the consent form and agreed with the Mei Jau Health Management Institute for using the information without personal identification for research purposes. Adult women aged 20–45 years with dyslipidemia were recruited between 2001 and 2015. Women aged 20–45 years were included in this study because these women could be at premenopausal status, and menopause usually occurred between the ages of 45 and 55 years in Asian women [12]. The mean age at menopause for Taiwanese women was 50.2 years (standard deviation of 4.0 years) [13]. Dyslipidemia was defined as one of the following criteria: high TG ≥ 2.26 mmol/L (200 mg/dL), high TC ≥ 6.21 mmol/L (240 mg/dL), high LDL-C ≥ 4.14 mmol/L (160 mg/dL), and low high-density lipoprotein cholesterol (HDL-C) < 1.29 mmol/L (50 mg/dL) [14]. The flow chart diagram of subjects included in the study is presented in Figure 1. There were 377,124 individuals who visited the Mei Jau health screening centers between 2001 and 2015. After excluding 343,428 participants who were male, <20 years, >45 years, pregnant, lactating, not dyslipidemic, had a history of cancer, cirrhosis, infection, lung disease, or kidney disease which could be accompanied by anemia, or had missing data and 11,065 women who had duplicate data because of multiple entries for physical examination between 2001 and 2015, 22,631 women were finally included in this study.

### 2.2. Anthropometric and Biochemical Data

Body weight, height, and waist circumference were retrieved from the database. Body mass index (BMI) was calculated as follows: body weight (kg)/height in square meters (m^2^), and classified into BMI <18.5 kg/m^2^ (underweight), 18.5 kg/m^2^ ≤ BMI < 24 kg/m^2^ (normal weight), 24 kg/m^2^ ≤ BMI < 27 kg/m^2^ (overweight), and BMI ≥27 kg/m^2^ (obesity) for Taiwanese adults [10]. Central obesity was defined as waist circumference (WC) ≥80 cm for Taiwanese women [15].

Participants fasted overnight (≥8 h) before participating in physical examination, and blood samples were measured for red blood cells (RBC), hemoglobin (Hb), hematocrit (Hct), blood lipids such as TG, TC, LDL-C, HDL-C, and C-reactive protein (CRP) [16,17]. All measurements of blood tests were obtained from the central laboratory of the Mei Jau Health Management Institute. Anemia was categorized into mild (Hb 11–11.9 g/dL), moderate (Hb 8–10.9 g/dL), and severe anemia (Hb < 8 g/dL) for women based on the World Health Organization classification [18].

### 2.3. Dietary Assessment

Dietary habits of participants were evaluated using a self-reported food frequency questionnaire (FFQ) validated by the Mei Jau Health Management Institute [11,15]. Fourteen food groups were analyzed in this study from twenty-two food groups of the FFQ. A plant diet consisted of 8 food groups: rice and flour products, whole grains, rhizomes, bread, light-colored vegetables, dark-colored vegetables, beans and legumes, and fruits. An animal diet consisted of 6 food groups: milk, dairy products, eggs, meat (such as chicken, duck, beef, lamb, and pork), organ meats (such as heart, intestines, kidney, and liver), and seafood. The dietary score for each food group was assigned from 1 to 5, from the lowest to the highest intake frequency [11]. The dietary scores for each individual were summed up for each food group according to the score of intake frequency. The minimum and maximum dietary scores ranged from 8 to 40 for the plant diet and from 6 to 30 for the animal diet. The median score of the participants was 16 for the plant diet and 12 for the animal diet. The median score was used to define high (≥median) or low (<median) intake [8]. Therefore, the dietary patterns were classified into low plant (LP) diet and low animal (LA) diet (LP + LA), low plant diet and high animal (HA) diet (LP + HA), high plant (HP) diet and low animal diet (HP + LA), and high plant diet and high animal diet (HP + HA) patterns.

### 2.4. Other Variables

Other variables of demographic data, lifestyle, and medical history as covariates were collected from self-reported questionnaires. Demographic and lifestyle data such as age, smoking, daily sleep duration, physical activity frequency, and vegetarian status were obtained when the subjects visited the Mei Jau health screening center before the physical examination. The age groups were stratified into 3 subgroups (20–30, 31–40, and 41–45 years) based on a 10-year class interval with a range of 20–45 years old. Smoking status was defined as no or yes for the current smoker. Daily sleep duration reflected the total daily sleep hours reported by the participants and was classified into short (<6 h), normal (6–8 h), and long (>8 h). Physical activity frequency was categorized into four levels: no (none or rarely), low (once a week), moderate (once every 2–3 days or once a day), and high (2–3 times a day). Exercise intensity was categorized as light (gardening, sweeping, mopping, golfing, baseball, calisthenics, dancing, or slow speed cycling), moderate (basketball, volleyball, table tennis, badminton, vigorous dancing, casual swimming, or fast walking), or vigorous intensity (jogging ≥ 8 m/h, running ≥ 12 m/h, climbing stairs, swimming, rope skipping, speed skating, or boat racing). Vegetarian status was defined as eating meatless meals daily and was categorized as no or yes for the current vegetarian.

### 2.5. Statistical Analysis

Statistical analysis was conducted using SAS version 9.4 (SAS Institute, Inc., Cary, NC, USA). The number (percentage) and the mean ± standard deviation, respectively, are presented for the categorical and continuous variables. The Kruskal–Wallis test for non-normal distribution was used for comparing multiple groups, and the chi-square test was performed for comparing categorical variables.

The multivariable logistic regression analysis was performed to identify the correlations between lifestyle, anthropometric, or dietary variables and the presence of anemia or the severity of anemia (mild vs. moderate to severe), and the data were expressed as odds ratios (ORs) and 95% confidence intervals (95% CIs). The association between dietary patterns and biochemical variables among dyslipidemic women was analyzed using the linear regression analysis, and the data were expressed as β coefficient and 95% CIs. The covariates were adjusted in different models. Model 1 was not adjusted, model 2 was adjusted for age, current smoker, sleep duration, physical activity frequency, and current vegetarian, and model 3 was adjusted for covariates in model 2 plus BMI and WC. The statistical significance was considered when the *p*-value was less than 0.05.

## 3. Results

### 3.1. Characteristics of the Participants with Different Dietary Patterns

Characteristics of dyslipidemic women with different dietary patterns are shown in Table 1. Among 22,631 dyslipidemic women, 26.6%, 19.7%, 19.2%, and 34.5% subjects consumed the LP + LA, LP + HA, HP + LA, and HP + HA patterns, respectively. The dyslipidemic women in this study had higher proportions of 31–40 years age group (48.9%), non-smokers (92.5%), normal sleep duration (77.8%), low physical activity frequency (68.5%), non-vegetarians (98.5%), normal BMI (59.2%), normal WC (98.5%), non-anemia (86.1%), and low HDL-C (75.7%). The prevalence of overweight, obesity, and central obesity was 18.6%, 14.1%, and 1.5%, respectively. Among dyslipidemic women, 13.9% of women had anemia, and 8.2% and 5.7% of women had mild or moderate to severe anemia, respectively. The mean Hb level of individuals was 13.1 ± 1.2 g/dL (range 4.2–17.6 g/dL). The prevalence of dyslipidemia for high TG, high TC, high LDL-C, and low HDL-C was 13.7%, 19.9%, 14.7%, and 75.7%, respectively.

Subjects consuming the LP + LA pattern were more likely to have short daily sleep duration (<6 h), low physical activity frequency (once a week), and be underweight compared to those with other dietary patterns. Subjects consuming the LP + HA pattern were more likely to be younger, current smokers, non-vegetarians, and non-anemic, and have higher levels of TC, LDL-C, RBC, Hb, Hct, and CRP. Subjects consuming the HP + LA pattern were more likely to be older and have higher proportions of non-smokers, vegetarians, moderate to high physical activity frequency, normal BMI, anemia, and low HDL-C, but less likely to have obese BMI, central obesity, high TC, and high LDL-C. Subjects consuming the HP + HA pattern were more likely to have long daily sleep duration (>8 h), overweight, obese BMI, central obesity, and higher RBC levels.

### 3.2. Association between Lifestyle, Anthropometric, or Dietary Variables and Anemia

The association between lifestyle, anthropometric, or dietary variables and the presence of anemia is demonstrated in Table 2. There were three models: model 1 was not adjusted, model 2 was adjusted for age, current smoker, sleep duration, physical activity frequency, and current vegetarian, and model 3 was adjusted for covariates in model 2 plus body mass index and waist circumference. Results in all models demonstrated that dyslipidemic women who were aged 31–45 years (model 3 OR: 1.22–1.62, 95% CI: 1.10–1.45, 1.36–1.81, *p* < 0.001) or current vegetarians (model 3 OR: 1.73, 95% CI: 1.34–2.24, *p* < 0.001) were correlated with higher odds of anemia. Only in model 3, dyslipidemic women who were underweight (OR: 1.22, 95% CI: 1.07–1.40, *p* < 0.05) were positively correlated with the presence of anemia. However, data in all models showed that subjects who were current smokers (model 3 OR: 0.64, 95% CI: 0.54–0.76, *p* < 0.001) or obese (model 3 OR: 0.71, 95% CI: 0.59–0.85, *p* < 0.001) were associated with lower odds of anemia. Only in model 1, subjects with central obesity were inversely associated with the presence of anemia (OR: 0.77, 95% CI: 0.69–0.85, *p* < 0.001). In models 1 and 2, dyslipidemic women consuming the LP + HA or HP + HA pattern were negatively associated with the presence of anemia, but subjects who consumed the HP + LA pattern were positively associated with the presence of anemia. In model 3, dyslipidemic women consuming the LP + HA pattern were correlated with lower odds of anemia by 13% (OR: 0.87, 95% CI: 0.78–0.98, *p* < 0.05) compared to those consuming the LP + LA pattern. However, subjects consuming the HP + LA pattern were associated with higher odds of anemia by 14% (OR: 1.14, 95% CI: 1.02–1.28, *p* < 005).

### 3.3. Association between Lifestyle, Anthropometric, or Dietary Variables and the Severity of Anemia

The association between lifestyle, anthropometric, or dietary variables and the severity of anemia is illustrated in Table 3. There were three models mentioned previously. Results in all models showed that dyslipidemic women who were aged 31–45 years or current vegetarians were correlated with higher risks of mild to severe anemia. Only in model 3, underweight subjects were correlated with higher odds of mild to severe anemia (mild anemia OR: 1.20, 95% CI: 1.01–1.42, *p* < 0.05, moderate to severe anemia OR: 1.25, 95% CI: 1.01–1.54, *p* < 0.05). On the contrary, data in all models demonstrated that subjects who were current smokers or obese (except for model 2) were associated with lower risks of mild to severe anemia. In model 1, subjects consuming the LP + HA pattern were correlated with lower odds of moderate to severe anemia by 29% (OR: 0.71, 95% CI: 0.59–0.85, *p* < 0.001) compared to those consuming the LP + LA pattern. However, subjects consuming the HP + LA pattern were associated with higher odds of mild to severe anemia (mild anemia OR: 1.17, 95% CI: 1.02–1.34, *p* < 0.05, moderate to severe anemia OR: 1.33, 95% CI: 1.13–1.55, *p* < 0.001). After adjustment for covariates in models 2 and 3, subjects consuming the LP + HA pattern were correlated with lower odds of moderate to severe anemia by 24% (OR: 0.76, 95% CI: 0.63–0.64, 0.91–0.92, *p* < 0.01) compared to those consuming the LP + LA pattern. However, subjects consuming the HP + LA pattern were associated with an increased risk of moderate to severe anemia by 22% (OR: 1.22, 95% CI: 1.04–1.43, *p* < 0.05).

### 3.4. Association between Dietary Patterns and Biochemical Variables

The association between dietary patterns and biochemical variables is shown in Table 4. There were three models described above. Before adjustment in model 1, subjects consuming the LP + HA pattern were correlated with higher levels of RBC (β: 0.02, 95% CI: 0.01, 0.04, *p* < 0.01), Hb (β: 0.09, 95% CI: 0.04, 0.14, *p* < 0.001), Hct (β: 0.27, 95% CI: 0.14, 0.40, *p* < 0.001), TC (β: 0.10, 95% CI: 0.06, 0.15, *p* < 0.001), LDL-C (β: 0.06, 95% CI: 0.03, 0.10, *p* < 0.001), and HDL-C (β: 0.03, 95% CI: 0.01, 0.04, *p* < 0.001) compared to subjects consuming the LP + LA pattern. After adjustment in models 2 and 3 showed that women consuming the LP + HA pattern were correlated with higher levels of Hb (model 2 β: 0.07, 95% CI: 0.02, 0.12, *p* < 0.01, model 3 β: 0.06, 95% CI: 0.00, 0.11, *p* < 0.05), Hct (model 2 β: 0.23, 95% CI: 0.10, 0.36, *p* < 0.01, model 3 β: 0.18, 95% CI: 0.06, 0.31, *p* < 0.01), TC (model 2 β: 0.14, 95% CI: 0.09, 0.18, *p* < 0.001, model 3 β: 0.08, 95% CI: 0.08, 0.17, *p* < 0.001), LDL-C (model 2 β: 0.09, 95% CI: 0.06, 0.13, *p* < 0.001, model 3 β: 0.08, 95% CI: 0.05, 0.12, *p* < 0.001), and HDL-C (model 2 β: 0.03, 95% CI: 0.01, 0.04, *p* < 0.001, model 3 β: 0.04, 95% CI: 0.02, 0.05, *p* < 0.001) compared to women consuming the LP + LA pattern. However, data in all models showed that women consuming the HP + LA pattern were negatively associated with the levels of RBC (model 3 β: −0.02, 95% CI: −0.03, −0.00, *p* < 0.05), Hb (model 3 β: −0.09, 95% CI: −0.13, −0.03, *p* < 0.001), Hct (model 3 β: −0.20, 95% CI: −0.33, −0.07, *p* < 0.01), TC (model 3 β: −0.17, 95% CI: −0.21, −0.12, *p* < 0.001), LDL-C (model 3 β: −0.13, 95% CI: −0.17, −0.09, *p* < 0.001), HDL-C (model 3 β: −0.03, 95% CI: −0.04, −0.01, *p* < 0.001), and CRP (model 3 β: −2.56, 95% CI: −4.08, −1.11, *p* < 0.001). Women consuming the HP + HA pattern were positively correlated with Hct (β: 0.11, 95% CI: 0.00, 0.22, *p* < 0.05), TG (β: 0.06, 95% CI: 0.03, 0.09, *p* < 0.001) in model 2, and HDL-C levels (β: 0.02, 95% CI: 0.01, 0.03, *p* < 0.01) in model 3, but not associated with anemia biomarkers, other blood lipids, or CRP in all models.

## 4. Discussion

Our results demonstrated that dyslipidemic women aged 41–45 years were associated with a higher risk of moderate to severe anemia by 120% compared to those aged 20–30 years. Additionally, dyslipidemic women who consumed the HP + LA pattern tended to have a higher prevalence of anemia (16.9%). The reasons for poor iron status or anemia among women of reproductive age, especially those who consumed a high plant and low animal diet, could be possibly due to the menstrual loss of iron and/or inadequate anemia-related nutrient intake [4,19]. Female lacto-vegetarians had a higher prevalence of anemia and serum vitamin B_12_ deficiency compared to the healthy controls in Taiwan, indicating that low dietary vitamin B_12_ intake in female lacto-vegetarians could contribute to serum vitamin B_12_ deficiency and further anemia [19].

### 4.1. Anthropometric Data and Anemia

In our study, approximately one-third of women (32.7%) were overweight or obese. Obesity and iron deficiency anemia are major global health problems. Both under- and over-nutrition could be related to anemia. A previous study found that the prevalence of anemia for female adult vegetarians with normal, overweight, or obese BMI was 26.0%, 27.7%, and 30.0% in Malaysia, respectively, indicating that overweight or obese individuals could have a higher risk of anemia than those with normal weight [8]. The BMI values were negatively correlated with the levels of anemia biomarkers (mean corpuscular volume, mean corpuscular hemoglobin, and transferrin saturation) in non-pregnant women aged 25–49 years in South Africa, suggesting that obesity was associated with a higher risk of anemia [20]. Obese Mexican women aged 18–50 years were associated with a higher risk of iron deficiency by 92% compared to those with normal weight [21]. Similarly, female adolescents with overweight or obesity (≥85th percentile for BMI) were associated with a higher risk of iron deficiency by 132% compared to those with normal BMI (5th ≤ BMI percentile < 85th) [22].

A higher risk of iron deficiency anemia caused by obesity could be attributed to obesity-induced inflammation of dietary iron absorption [21,23] or iron metabolism [23,24]. Obese subjects had increased hepcidin levels which were correlated with low-grade systemic inflammation [23]. Lack of functional iron was associated with adipose tissue inflammation and increased secretion of hepcidin [24]. Elevated circulating hepcidin in response to inflammation or iron overload inhibits cellular iron export and consequently decreases serum iron levels for maintenance of systemic iron homeostasis [24]. Contrastingly, our study showed that obesity was correlated with a lower risk of mild to severe anemia. Consistent with our findings, adult women who were overweight or obese were found to have a lower risk of iron deficiency anemia compared to those with normal weight in Taiwan [25]. Hemoglobin levels were increased with increasing BMI values among Chinese adult women, suggesting that BMI values were inversely correlated with anemia [26]. Additionally, overweight or obese women had lower prevalence ratios of anemia compared to those with normal weight [26]. Our findings demonstrated that dyslipidemic women who were underweight were associated with a higher risk of mild to severe anemia by 20–25% compared to those with normal weight. Similarly, a previous study also found that being underweight was correlated with a higher risk of anemia among adult women in Bangladesh [27]. Women of reproductive age who were underweight in East Java, Indonesia were more likely to have iron-deficient erythropoiesis almost 4 times higher than those who were not underweight [28].

### 4.2. Dietary Patterns and Anemia

Our results found that dyslipidemic women consuming the LP + HA pattern were correlated with less anemia but higher cholesterol levels, whereas those consuming the HP + LA pattern were associated with more anemia but less obesity and lower cholesterol and CRP levels. The absorption of iron was approximately 10% and 18% for a vegetarian diet and a mixed Western diet with animal foods, respectively [29]. The animal source of iron such as heme-rich red meat can increase the bioavailability and solubility of heme iron [30]. The absorption of non-heme iron can be inhibited by the ingredients in plant foods such as phytate, tannin, and phosphate or by polyphenol-containing drinks such as black tea, herb tea, coffee, cocoa, and red wine [31,32]; in contrast, foods abundant in vitamin C can increase the solubility and absorption of non-heme iron [26,30], which may compensate poor bioavailability of non-heme iron.

Our results also found that obese women were less likely to have the HP + LA pattern which was the risk factor for anemia. This observation may explain that obese women had a lower risk of anemia in this study. A previous study has supported that the prevalence of anemia was linked to dietary habits [33]. The prevalence of anemia was higher in white premenopausal women who were vegetarians compared to those who were regular meat eaters (12.8% vs. 8.7%, *p* < 0.05) in the UK [33]. The dietary patterns could contribute to different consumption of nutrients which are correlated to erythropoiesis or the development of anemia. The major contribution of meat such as red meat, poultry, seafood, and organ meats to daily dietary intakes of iron and vitamin B_12_ was 4.3–5.2% and 19.7–34.4%, respectively, among adult women in six ethnic groups in the US [34], suggesting that an animal diet may play a substantial role in the supply of iron and vitamin B_12_. Iron, vitamin B_12_, and folate are predominant nutrients for erythropoiesis, and a lack of these nutrients could consequently lead to the development of anemia [35].

Our findings demonstrated that women consuming the LP + HA pattern were more likely to have higher Hb levels by 0.06 g/dL and Hct levels by 0.18%. The meta-analysis of 24 cross-sectional studies also found that serum ferritin levels in vegetarian adults were significantly lowered compared to those in non-vegetarian adults [36]. However, a plant diet has been reported to be associated with better health outcomes such as a lower BMI, better body compositions in lean body mass and body fat, decreased glycated Hb, lower blood lipids, lower all-cause mortality, and lower risks for obesity, cardiovascular or cardiometabolic disease, cerebrovascular disease, insulin resistance, type 2 diabetes mellitus, and certain types of cancer compared to an omnivore diet [5,37,38]. We also observed that dyslipidemic women consuming the HP + LA pattern were more likely to have better weight status and blood lipid profiles, but increase mild to severe anemia. Although a high plant diet was generally linked to better health outcomes, the form of nutrients such as heme iron in animal foods and non-heme iron in plant foods can influence the iron status and the risk of iron deficiency anemia. Non-heme iron in plant foods was less absorbed than heme iron, and the absorption of iron was dependent upon the balance between the inhibitors or enhancers of iron absorption and the iron status of the individual [39].

Concurrently, the long-term vegetarians may have insufficient intakes of certain essential nutrients which are not abundant in plant foods. Vegetarians mostly had adequate intakes of macro- and micronutrients throughout all stages of the life cycle, but had a lower status of n-3 fatty acids, vitamin B_12_, vitamin D, calcium, iron, and zinc because of less intake, decreased absorption, or lower bioavailability compared to those who were omnivorous [6]. A cross-sectional study in Belgium showed that the vegetarians had lower absolute intakes of energy, protein, saturated fat, monounsaturated fat, cholesterol, and sodium, but higher intakes of fiber, calcium, and iron compared to the omnivores [40]. Even though the vegetarians had a higher iron intake, they had lower serum ferritin levels than meat eaters due to less bioavailability of non-heme iron in plant foods [41]. Additionally, insufficient intake of animal foods could increase the risk of anemia, and low iron stores could lead to iron deficiency anemia [41].

### 4.3. Strengths and Limitations

The strength of this study was its relatively large sample size of dyslipidemic adult women. Additionally, dietary patterns combined two factors including the type (plant vs. animal food) and the intake (low vs. high). However, this study had several limitations. The self-reported FFQ used at the MJ health screening center only provided estimates of habitual food intake rather than the actual intake of nutrients or total calories. Additionally, the use of a self-administered questionnaire might initiate some potential self-reporting bias, which could be mitigated via collecting data by well-trained interviewers or via measuring objectively to increase the accuracy and consistency of the results. This retrospective study neither identified a causal relationship nor generalized to other populations, particularly non-Taiwanese ethnic groups. More factors including demographic and clinical characteristics, inclusion and exclusion criteria, and study setting or intervention study should be considered for better generalizability. The present study was constrained to determine the type of anemia because there were no relevant biochemical markers such as vitamin B_12_, folate, ferritin, and iron status in our available database. In addition, several factors such as genetic variations, consumption of supplements (iron or vitamin C), taking certain medication which may interfere with the results, diagnosed CVD, and menstruation condition (menopause) were not considered due to lack of information.

## 5. Conclusions

This study identifies four types of dietary patterns based on a low or high plant or animal diet. A low plant and high animal diet plays a role in preventing anemia development among dyslipidemic women in Taiwan. However, a low plant and high animal diet increase cholesterol-related variables. The findings could be the reference for public health implications to strengthen nutrition knowledge in a balanced diet for both plant and animal food intake among dyslipidemic adult women to decrease the risk of anemia. Dietary, lifestyle, and sociodemographic factors should be considered when developing anemia prevention programs. In addition, more research is needed to link dietary patterns to anemia in dyslipidemic patients.

## Figures and Tables

**Table 1:** Characteristics of dyslipidemic women aged 20–45 years (*n* = 22,631) with different dietary patterns.

Characteristic	Total (*n* = 22,631)	Dietary Pattern				*p*-Value ^3^
		LP ^1^ + LA ^1^ (*n* = 6021)	LP ^1^ + HA ^2^ (*n* = 4459)	HP ^2^ + LA ^1^ (*n* = 4333)	HP ^2^ + HA ^2^ (*n* = 7818)	
Age, years						
20–30	5274 (23.3)	1495 (24.8)	1348 (30.2)	717 (16.5)	1714 (21.9)	<0.0001
31–40	11,061 (48.9)	2827 (47.0)	2191 (49.2)	2084 (48.1)	3959 (50.7)	
41–45	6296 (27.8)	1699 (28.2)	920 (20.6)	1532 (35.4)	2145 (27.4)	
Current smoker, *n* (%)						<0.0001
No	20,927 (92.5)	5436 (90.3)	3976 (89.2)	4153 (95.8)	7362 (94.2)	
Yes	1704 (7.5)	585 (9.7)	483 (10.8)	180 (4.2)	456 (5.8)	
Daily sleep duration, *n* (%)						<0.0001
Short (<6 h)	4475 (19.8)	1318 (21.9)	925 (20.8)	813 (18.2)	1419 (18.1)	
Normal (6–8 h)	17,603 (77.8)	4562 (75.8)	3417 (76.6)	3433 (79.2)	6191 (79.2)	
Long (>8 h)	553 (2.4)	141 (2.3)	117 (2.6)	87 (2.0)	208 (2.7)	
Physical activity frequency,^4^ *n* (%)						<0.0001
No	291 (1.3)	73 (1.2)	47 (1.1)	53 (1.2)	118 (1.5)	
Low	15,495 (68.5)	4542 (75.4)	3190 (71.5)	2771 (64.0)	4992 (63.8)	
Moderate	4696 (20.7)	1016 (16.9)	883 (19.8)	1002 (23.1)	1795 (23.0)	
High	2149 (9.5)	390 (6.5)	339 (7.6)	507 (11.7)	913 (11.7)	
Current vegetarian, *n* (%)						<0.0001
No	22,292 (98.5)	5948 (98.8)	4446 (99.7)	4119 (95.1)	7779 (99.5)	
Yes	339 (1.5)	73 (1.2)	13 (0.3)	214 (4.9)	39 (0.5)	
BMI,^5^ *n* (%)						<0.0001
BMI <18.5 kg/m^2^	1840 (8.1)	551 (9.2)	368 (8.3)	361 (8.3)	560 (7.2)	
18.5 kg/m^2^ ≤ BMI < 24 kg/m^2^	13,387 (59.2)	3632 (60.3)	2579 (57.8)	2668 (61.6)	4508 (57.6)	
24 kg/m^2^ ≤ BMI <27 kg/m^2^	4219 (18.6)	1086 (18.0)	835 (18.7)	799 (18.4)	1499 (19.2)	
BMI ≥ 27 kg/m^2^	3185 (14.1)	752 (12.5)	677 (15.2)	505 (11.7)	1251 (16.0)	
Waist circumference,^6^ *n* (%)						<0.0001
Normal	18,325 (98.5)	4984 (82.8)	3535 (79.3)	3639 (84.0)	6167 (78.9)	
Central obesity	4306 (1.5)	1037 (17.2)	924 (20.7)	694 (16.0)	1651 (21.1)	
Anemia,^7^ *n* (%)						<0.0001
No	19,499 (86.1)	5170 (85.9)	3924 (88.0)	3601 (83.1)	6804 (87.0)	
Mild	1847 (8.2)	498 (8.3)	346 (7.8)	406 (9.4)	597 (7.7)	
Moderate to severe	1285 (5.7)	353 (5.8)	189 (4.2)	326 (7.5)	417 (5.3)	
Dyslipidemia,^8^ *n* (%)						
High TG	3094 (13.7)	769 (12.8)	623 (14.0)	597 (13.8)	1105 (14.1)	0.1152
High TC	4496 (19.9)	1220 (20.3)	993 (22.3)	711 (16.4)	1572 (20.1)	<0.0001
High LDL-C	3322 (14.7)	940 (15.6)	731 (16.4)	516 (11.9)	1135 (14.5)	<0.0001
Low HDL-C	17,126 (75.7)	4565 (75.8)	3229 (72.4)	3443 (79.5)	5889 (75.3)	<0.0001
Biochemical data						
RBC, 10^6^/μL	4.5 ± 0.4	4.5 ± 0.4	4.6 ± 0.4	4.5 ± 0.4	4.6 ± 0.4	<0.0001
Hb, g/dL	13.1 ± 1.2	13.1 ± 1.3	13.2 ± 1.2	12.9 ± 1.4	13.1 ± 1.2	<0.0001
Hct, %	39.1 ± 3.3	39.1 ± 3.3	39.4 ± 3.1	38.8 ± 3.6	39.2 ± 3.2	<0.0001
C-reactive protein, nmol/L	23.3 ± 38.1	23.7 ± 42.2	25.0 ± 41.2	20.6 ± 31.4	23.6 ± 36.2	<0.0001

^1^ The dietary scores of the high plant diet (HP) and high animal diet (HA) are greater than or equal to the median. ^2^ The dietary scores of low plant diet (LP) and low animal diet (LA) are less than the median. ^3^ The *p*-value was analyzed using a chi-square test for categorical variables and a Mann–Whitney *U* test for continuous variables. ^4^ Physical activity frequency was categorized into 4 levels: no (none or rarely), low (once a week), moderate (once every 2–3 days or once a day), and high (2–3 times a day). ^5^ Body mass index was categorized into BMI < 18.5 kg/m^2^ (underweight), 18.5 kg/m^2^ ≤ BMI < 24 kg/m^2^ (normal weight), 24 kg/m^2^ ≤ BMI < 27 kg/m^2^ (overweight), and BMI ≥ 27 kg/m^2^ (obesity). ^6^ Waist circumference was categorized into normal (<80 cm) and central obesity (≥80 cm) for women in Taiwan. ^7^ Normal Hb level is 12–15.5 g/dL for women. Anemia was categorized into mild (Hb 11–11.9 g/dL), moderate (Hb 8–10.9 g/dL), and severe anemia (Hb < 8 g/dL). ^8^ Dyslipidemia was defined as high TG ≥ 2.26 mmol/L (200 mg/dL), high TC ≥ 6.21 mmol/L (240 mg/dL), high LDL-C ≥ 4.14 mmol/L (160 mg/dL), and low HDL-C < 1.29 mmol/L (50 mg/dL). Data are expressed as number (percentage) or mean ± standard deviation, respectively, for categorical or continuous variables. LP: low plant diet, LA: low animal diet, HA: high animal diet, HP: high plant diet, BMI: body mass index, TG: triglycerides, TC: total cholesterol, LDL-C: low-density lipoprotein cholesterol, HDL-C: high-density lipoprotein cholesterol, RBC: red blood cells, Hb: hemoglobin, Hct: hematocrit.

**Table 2:** Association between lifestyle, anthropometric, or dietary variables and the presence of anemia among dyslipidemic women aged 20–45 years (*n* = 22,631) in Taiwan.

Variable	Model 1	Model 2	Model 3
	OR (95% CI)	OR (95% CI)	OR (95% CI)
Age, years			
20–30	1	1	1
31–40	1.23 (1.11–1.36) ***	1.19 (1.08–1.32) ***	1.22 (1.10–1.36) ***
41–45	1.63 (1.46–1.82) ***	1.55 (1.38–1.73) ***	1.62 (1.45–1.81) ***
Current smoker			
No	1	1	1
Yes	0.60 (0.51–0.72) ***	0.65 (0.55–0.77) ***	0.64 (0.54–0.76) ***
Daily sleep duration			
Normal (6–8 h)	1	1	1
Short (<6 h)	1.01 (0.92–1.11)	1.01 (0.92–1.11)	1.03 (0.94–1.14)
Long (>8 h)	0.87 (0.67–1.13)	0.91 (0.70–1.18)	0.91 (0.71–1.18)
Physical activity frequency ^1^			
No	1	1	1
Low	1.29 (0.89–1.87)	1.35 (0.93–1.96)	1.34 (0.93–1.95)
Moderate	1.31 (0.90–1.91)	1.34 (0.92–1.96)	1.33 (0.91–1.94)
High	1.35 (0.92–1.98)	1.33 (0.90–1.96)	1.31 (0.88–1.93)
Current vegetarian			
No	1	1	1
Yes	2.01 (1.56–2.58) ***	1.73 (1.34–2.24) ***	1.73 (1.34–2.24) ***
BMI,^2^ kg/m^2^			
18.5 ≤ BMI < 24	1	N/A	1
BMI <18.5	1.10 (0.96–1.26)	N/A	1.22 (1.07–1.40) *
24 ≤ BMI < 27	0.94 (0.85–1.04)	N/A	0.92 (0.83–1.03)
BMI ≥ 27	0.69 (0.61–0.78) ***	N/A	0.71 (0.59–0.85) ***
Waist circumference ^3^			
Normal	1	N/A	1
Central obesity	0.77 (0.69–0.85) ***	N/A	0.96 (0.83–1.12)
Dietary pattern ^4^			
LP + LA	1	1	1
LP + HA	0.83 (0.74–0.93) **	0.86 (0.77–0.97) *	0.87 (0.78–0.98) *
HP + LA	1.23 (1.11–1.37) ***	1.15 (1.03–1.28) *	1.14 (1.02–1.28) *
HP + HA	0.91 (0.82–0.99) *	0.89 (0.81–0.99) *	0.91 (0.83–1.01)

^1^ Physical activity frequency was categorized into 4 levels: no (none or rarely), low (once a week), moderate (once every 2–3 days or once a day), and high (2–3 times a day). ^2^ Body mass index was categorized into BMI < 18.5 kg/m^2^ (underweight), 18.5 kg/m^2^ ≤ BMI < 24 kg/m^2^ (normal weight), 24 kg/m^2^ ≤ BMI < 27 kg/m^2^ (overweight), and BMI ≥ 27 kg/m^2^ (obesity). ^3^ Waist circumference was categorized into normal (<80 cm) and central obesity (≥80 cm) for women in Taiwan. ^4^ The dietary scores of high plant diet (HP) and high animal diet (HA) were greater than or equal to the median, and those of low plant diet (LP) and low animal diet (LA) were less than the median. Data were analyzed by multivariable logistic regression, and expressed as OR (95% CI). Model 1 was not adjusted, model 2 was adjusted for age, current smoker, sleep duration, physical activity frequency, and current vegetarian, and model 3 was adjusted for covariates in model 2 plus body mass index and waist circumference. Anemia was defined as hemoglobin < 12 g/dL for women. * *p* < 0.05, ** *p* < 0.01, *** *p* < 0.001. OR: odds ratio, CI: confidence interval, BMI: body mass index, N/A: not applicable, LP: low plant diet, LA: low animal diet, HA: high animal diet, HP: high plant diet.

**Table 3:** Association between lifestyle, anthropometric, or dietary variables and mild or moderate to severe anemia among dyslipidemic women aged 20–45 years (*n* = 22,631) in Taiwan.

Variable	Model 1		Model 2		Model 3	
	Mild Anemia (*n* = 1847) OR (95% CI)	Moderate to Severe Anemia (*n* = 1285) OR (95% CI)	Mild Anemia (*n* = 1847) OR (95% CI)	Moderate to Severe Anemia (*n* = 1285) OR (95% CI)	Mild Anemia (*n* = 1847) OR (95% CI)	Moderate to Severe Anemia (*n* = 1285) OR (95% CI)
Age, years						
20–30	1	1	1	1	1	1
31–40	1.18 (1.04–1.34) **	1.31 (1.11–1.541) **	1.16 (1.02–1.31) *	1.27 (1.07–1.49) **	1.19 (1.05–1.35) **	1.29 (1.09–1.53) **
41–45	1.29 (1.12–1.48) ***	2.27 (1.92–2.67) ***	1.23 (1.07–1.41) **	2.13 (1.80–2.52) ***	1.29 (1.13–1.49) ***	2.20 (1.86–2.61) ***
Current smoker						
No	1	1	1	1	1	1
Yes	0.61 (0.49–0.75) ***	0.59 (0.46–0.77) ***	0.63 (0.51–0.78) ***	0.67 (0.52–0.87) **	0.63 (0.50–0.78) ***	0.67 (0.52–0.87) **
Daily sleep duration						
Normal (6–8 h)	1	1	1		1	
Short (<6 h)	0.97 (0.86–1.09)	1.08 (0.94–1.24)	0.97 (0.86–1.10)	1.06 (0.92–1.22)	1.00 (0.89–1.14)	1.08 (0.94–1.24)
Long (>8 h)	0.99 (0.73–1.35)	0.69 (0.45–1.07)	1.04 (0.76–1.41)	0.72 (0.47–1.11)	1.04 (0.76–1.42)	0.72 (0.47–1.12)
Physical activity frequency ^1^						
No	1	1	1	1	1	
Low	1.35 (0.83–2.18)	1.23 (0.71–2.11)	1.35 (0.83–2.19)	1.34 (0.78–2.32)	1.35 (0.83–2.19)	1.34 (0.78–2.31)
Moderate	1.33 (0.82–2.17)	1.29 (0.74–2.23)	1.33 (0.81–2.17)	1.36 (0.78–2.37)	1.31 (0.80–2.14)	1.35 (0.78–2.35)
High	1.59 (0.97–2.62)	1.03 (0.58–1.84)	1.57 (0.95–2.59)	1.02 (0.57–1.81)	1.53 (0.93–2.53)	1.01 (0.57–1.79)
Current vegetarian						
No	1	1	1	1	1	1
Yes	1.91 (1.39–2.63) ***	2.16 (1.16–3.07) ***	1.73 (1.25–2.39) ***	1.73 (1.21–2.48) **	1.73 (1.25–2.39) ***	1.74 (1.21–2.49) **
BMI,^2^ kg/m^2^						
18.5 ≤ BMI < 24	1	1	N/A	N/A	1	1
BMI <18.5	1.13 (0.95–1.33)	1.07 (0.87–1.32)	N/A	N/A	1.20 (1.01–1.42) *	1.25 (1.01–1.54) *
24 ≤ BMI < 27	0.84 (0.74–0.96) **	1.09 (0.95–1.27)	N/A	N/A	0.85 (0.74–0.98) *	1.01 (0.86–1.19)
BMI ≥ 27	0.65 (0.56–0.77) ***	0.74 (0.61–0.89) **	N/A	N/A	0.72 (0.58–0.91) **	0.70 (0.54–0.91) **
Waist circumference ^3^						
Normal	1	1	N/A	N/A	1	
Central Obesity	0.71 (0.62–0.81) ***	0.86 (0.75–1.001)	N/A	N/A	0.89 (0.74, 1.09)	1.05 (0.84, 1.29)
Dietary pattern ^4^						
LP + LA	1	1	1	1	1	1
LP + HA	0.92 (0.79–1.06)	0.71 (0.59–0.85) ***	0.93 (0.81–1.08)	0.76 (0.63–0.91) **	0.95 (0.82, 1.09)	0.76 (0.64, 0.92) **
HP + LA	1.17 (1.02–1.34) *	1.33 (1.13–1.55) ***	1.09 (0.95–1.26)	1.22 (1.04–1.43) *	1.09 (0.95, 1.25)	1.22 (1.04, 1.43) *
HP + HA	0.91 (0.80–1.03)	0.89 (0.78–1.04)	0.89 (0.79–1.01)	0.91 (0.78–1.01)	0.91 (0.80, 1.03)	0.92 (0.79, 1.06)

^1^ Physical activity frequency was categorized into 4 levels: no (none or rarely), low (once a week), moderate (once every 2–3 days or once a day), and high (2–3 times a day). ^2^ Body mass index was categorized into BMI < 18.5 kg/m^2^ (underweight), 18.5 kg/m^2^ ≤ BMI < 24 kg/m^2^ (normal weight), 24 kg/m^2^ ≤ BMI < 27 kg/m^2^ (overweight), and BMI ≥ 27 kg/m^2^ (obesity). ^3^ Waist circumference was categorized into normal (<80 cm) and central obesity (≥80 cm) for women in Taiwan. ^4^ The dietary scores of the high plant diet (HP) and high animal diet (HA) were greater than or equal to the median, and those of low plant diet (LP) and low animal diet (LA) were less than the median. Data were analyzed by multivariable logistic regression and expressed as OR (95% CI). Model 1 was not adjusted, model 2 was adjusted for age, current smoker, sleep duration, physical activity frequency, and current vegetarian, and model 3 was adjusted for covariates in model 2 plus body mass index and waist circumference. Mild anemia was defined as hemoglobin 11–11.9 g/dL, and moderate to severe anemia was defined as hemoglobin <8–10.9 g/dL. * *p* < 0.05, ** *p* < 0.01, *** *p* < 0.001. OR: odds ratio, CI: confidence interval, BMI: body mass index, N/A: not applicable, LP: low plant diet, LA: low animal diet, HA: high animal diet, HP: high plant diet.

**Table 4:** Association between dietary patterns and biochemical variables among dyslipidemic women aged 20–45 years (*n* = 22,631) in Taiwan.

Variable	LP + HA			HP + LA			HP + HA		
	Model 1 β (95% CI)	Model 2 β (95% CI)	Model 3 β (95% CI)	Model 1 β (95% CI)	Model 2 β (95% CI)	Model 3 β (95% CI)	Model 1 β (95% CI)	Model 2 β (95% CI)	Model 3 β (95% CI)
RBC, 10^6^/μL	0.02 (0.01, 0.04) **	0.02 (0.00, 0.04)	0.01 (−0.00, 0.03)	−0.02 (−0.04, −0.01) **	−0.02 (−0.04, −0.01) **	−0.02 (−0.03, −0.00) *	0.01 (−0.01, 0.02)	0.01 (−0.01, 0.02)	−0.00 (−0.01, 0.02)
Hb, g/dL	0.09 (0.04, 0.14) ***	0.07 (0.02, 0.12) **	0.06 (0.00, 0.11) *	−0.13 (−0.18, −0.08) ***	−0.09 (−0.14, −0.04) ***	−0.09 (−0.13, −0.03) ***	0.03 (−0.01, 0.07)	0.04 (−0.00, 0.08)	0.03 (−0.01, 0.07)
Hct, %	0.27 (0.14, 0.40) ***	0.23 (0.10, 0.36) **	0.18 (0.06, 0.31) **	−0.31 (−0.44, −0.18) ***	−0.22 (−0.35, −0.09) **	−0.20 (−0.33, −0.07) **	0.09 (−0.02, 0.20)	0.11 (0.00, 0.22) *	0.06 (−0.04, 0.17)
TG, mmol/L	0.02 (−0.01, 0.05)	0.05 (0.01, 0.08) **	0.02 (−0.01, 0.05)	0.01 (−0.02, 0.04)	0.00 (−0.03, 0.03)	0.00 (−0.03, 0.04)	0.05 (0.02, 0.07) *	0.06 (0.03, 0.09) ***	0.02 (−0.00, 0.05)
TC, mmol/L	0.10 (0.06, 0.15) ***	0.14 (0.09, 0.18) ***	0.08 (0.08, 0.17) ***	−0.14 (−0.19, −0.10) ***	−0.17 (−0.21, −0.12) ***	−0.17 (−0.21, −0.12) ***	0.01 (−0.03, 0.05)	0.00 (−0.03, 0.04)	−0.00 (−0.04, 0.03)
LDL-C, mmol/L	0.06 (0.03, 0.10) ***	0.09 (0.06, 0.13) ***	0.08 (0.05, 0.12) ***	−0.12 (−0.16, −0.08) ***	−0.13 (−0.17, −0.09) ***	−0.13 (−0.17, −0.09) ***	−0.02 (−0.05, 0.02)	−0.02 (−0.05, 0.01)	−0.02 (−0.06, 0.01)
HDL-C, mmol/L	0.03 (0.01, 0.04) ***	0.03 (0.01, 0.04) ***	0.04 (0.02, 0.05) ***	−0.03 (−0.04, −0.01) ***	−0.03 (−0.04, −0.01) ***	−0.03 (−0.04, −0.01) ***	0.01 (−0.00, 0.02)	0.01 (−0.00, 0.02)	0.02 (0.01, 0.03) **
CRP, nmol/L	1.31 (−0.17, 2.78)	1.43 (−0.04, 2.91)	−0.17 (−1.74, 1.39)	−3.07 (−4.55, −1.58) ***	−2.69 (−4.19, −1.20) ***	−2.56 (−4.08, −1.11) ***	−0.11 (−1.39, 1.17)	0.27 (−1.02, 1.55)	−1.07 (−2.31, 0.18)

The low plant diet + low animal diet pattern was the reference group. Model 1 was not adjusted, model 2 was adjusted for age, current smoker, sleep duration, physical activity frequency, and current vegetarian, and model 3 was adjusted for covariates in model 2 plus body mass index and waist circumference. * *p* < 0.05, ** *p* < 0.01, *** *p* < 0.001. LP: low plant diet, HA: high animal diet, HP: high plant diet, LA: low animal diet, CI: confidence interval, RBC: red blood cells, Hb: hemoglobin, Hct: hematocrit, TG: triglycerides, TC: total cholesterol, LDL-C: low-density lipoprotein cholesterol, HDL-C: high-density lipoprotein cholesterol, CRP: C-reactive protein.

## Data Availability

The Mei Jau Health Management Institute provides information supporting the results of this study but is prescribed for research use only. The questionnaire and data are not open to the public. The questionnaire and data are accessible from the authors upon appropriate request and with approval of the Mei Jau Health Management Institute.

## References

[1] Health Promotion Administration, Ministry of Health and Welfare (2020). 2020 Health Promotion Administration Annual Report.

[2] Nelson R.H. (2013). Hyperlipidemia as a risk factor for cardiovascular disease. Prim. Care.

[3] Lin C.-F., Chang Y.-H., Chien S.-C., Lin Y.-H., Yeh H.-Y. (2018). Epidemiology of dyslipidemia in the Asia Pacific region. Int. J. Gerontol..

[4] Lin L.Y., Hsu C.Y., Lee H.A., Wang W.H., Kurniawan A.L., Chao J.C. (2019). Dietary patterns in relation to components of dyslipidemia and fasting plasma glucose in adults with dyslipidemia and elevated fasting plasma glucose in Taiwan. Nutrients.

[5] Igl W., Kamal-Eldin A., Johansson A., Liebisch G., Gnewuch C., Schmitz G., Gyllensten U. (2013). Animal source food intake and association with blood cholesterol, glycerophospholipids and sphingolipids in a northern Swedish population. Int. J. Circumpolar Health.

[6] Craig W.J., Mangels A.R. (2009). Position of the American Dietetic Association: Vegetarian diets. J. Am. Diet. Assoc..

[7] Coad J., Pedley K. (2014). Iron deficiency and iron deficiency anemia in women. Scand. J. Clin. Lab. Investig..

[8] Chai Z.F., Gan W.Y., Chin Y.S., Ching Y.K., Appukutty M. (2019). Factors associated with anemia among female adult vegetarians in Malaysia. Nutr. Res. Pract..

[9] Capuano E., Oliviero T., van Boekel M.A.J.S. (2018). Modeling food matrix effects on chemical reactivity: Challenges and perspectives. Crit. Rev. Food Sci. Nutr..

[10] Kurniawan A.L., Hsu C.Y., Rau H.H., Lin L.Y., Chao J.C. (2019). Association of kidney function-related dietary pattern, weight status, and cardiovascular risk factors with severity of impaired kidney function in middle-aged and older adults with chronic kidney disease: A cross-sectional population study. Nutr. J..

[11] Muga M.A., Owili P.O., Hsu C.Y., Rau H.H., Chao J.C. (2016). Association between dietary patterns and cardiovascular risk factors among middle-aged and elderly adults in Taiwan: A population-based study from 2003 to 2012. PLoS ONE.

[12] Wang M., Gong W., Hu R., Wang H., Guo Y., Bian Z., Lv J., Chen Z., Li L., Yu M. (2018). Age at natural menopause and associated factors in adult women: Findings from the China Kadoorie Biobank study in Zhejiang rural area. PLoS ONE.

[13] Shen T.-Y., Strong C., Yu T. (2020). Age at menopause and mortality in Taiwan: A cohort analysis. Maturitas.

[14] Harikumar K., Althaf S.A., Kumar B.K., Ramunaik M., Suvarna C. (2013). A review on hyperlipidemic. Nov. Sci. Int. J. Pharm. Sci..

[15] Syauqy A., Hsu C.Y., Rau H.H., Chao J.C. (2018). Association of dietary patterns with components of metabolic syndrome and inflammation among middle-aged and older adults with metabolic syndrome in Taiwan. Nutrients.

[16] Avila F., Echeverria G., Perez D., Martinez C., Strobel P., Castillo O., Villaroel L., Mezzano D., Rozowski J., Urquiaga I. (2015). Serum ferritin is associated with metabolic syndrome and red meat consumption. Oxid. Med. Cell. Longev..

[17] Pedersen K.M., Colak Y., Ellervik C., Hasselbalch H.C., Bojesen S.E., Nordestgaard B.G. (2019). Smoking and increased white and red blood cells. Arterioscler. Thromb. Vasc. Biol..

[18] World Health Organiztion (1972). Nutritional anaemias. Report of a WHO group of experts. World Health Organ. Tech. Rep. Ser..

[19] Lee Y.P., Loh C.H., Hwang M.J., Lin C.P. (2021). Vitamin B12 deficiency and anemia in 140 Taiwanese female lacto-vegetarians. J. Med. Assoc..

[20] Jordaan E.M., van den Berg V.L., van Rooyen F.C., Walsh C.M. (2020). Obesity is associated with anaemia and iron deficiency indicators among women in the rural Free State, South Africa. S. Afr. J. Clin. Nutr..

[21] Cepeda-Lopez A.C., Osendarp S.J., Melse-Boonstra A., Aeberli I., Gonzalez-Salazar F., Feskens E., Villalpando S., Zimmermann M.B. (2011). Sharply higher rates of iron deficiency in obese Mexican women and children are predicted by obesity-related inflammation rather than by differences in dietary iron intake. Am. J. Clin. Nutr..

[22] Tussing-Humphreys L.M., Liang H., Nemeth E., Freels S., Braunschweig C.A. (2009). Excess adiposity, inflammation, and iron-deficiency in female adolescents. J. Am. Diet. Assoc..

[23] Cepeda-Lopez A.C., Aeberli I., Zimmermann M.B. (2010). Does obesity increase risk for iron deficiency? A review of the literature and the potential mechanisms. Int. J. Vitam. Nutr. Res..

[24] Aigner E., Feldman A., Datz C. (2014). Obesity as an emerging risk factor for iron deficiency. Nutrients.

[25] Chang J.S., Chen Y.C., Owaga E., Palupi K.C., Pan W.H., Bai C.H. (2014). Interactive effects of dietary fat/carbohydrate ratio and body mass index on iron deficiency anemia among Taiwanese women. Nutrients.

[26] Qin Y., Melse-Boonstra A., Pan X., Yuan B., Dai Y., Zhao J., Zimmermann M.B., Kok F.J., Zhou M., Shi Z. (2013). Anemia in relation to body mass index and waist circumference among Chinese women. Nutr. J..

[27] Ghose B., Yaya S., Tang S. (2016). Anemia status in relation to body mass index among women of childbearing age in Bangladesh. Asia Pac. J. Public Health.

[28] Sumarmi S., Puspitasari N., Handajani R., Wirjatmadi B. (2016). Underweight as a risk factor for iron depletion and iron-deficient erythropoiesis among young women in rural areas of East Java, Indonesia. Malays. J. Nutr..

[29] National Health and Medical Research Council (2017). Nutrient Reference Values for Australia and New Zealand Including Recommended Dietary Intakes.

[30] West A.R., Oates P.S. (2008). Mechanisms of heme iron absorption: Current questions and controversies. World J. Gastroenterol..

[31] Hazell T. (1988). Relating food composition data to iron availability from plant foods. Eur. J. Clin. Nutr..

[32] Hurrell R.F., Reddy M., Cook J.D. (1999). Inhibition of non-haem iron absorption in man by polyphenolic-containing beverages. Br. J. Nutr..

[33] Tong T.Y.N., Key T.J., Gaitskell K., Green T.J., Guo W., Sanders T.A., Bradbury K.E. (2019). Hematological parameters and prevalence of anemia in white and British Indian vegetarians and nonvegetarians in the UK Biobank. Am. J. Clin. Nutr..

[34] Sharma S., Sheehy T., Kolonel L.N. (2013). Contribution of meat to vitamin B_12_, iron and zinc intakes in five ethnic groups in the USA: Implications for developing food-based dietary guidelines. J. Hum. Nutr. Diet..

[35] Koury M.J., Ponka P. (2004). New insights into erythropoiesis: The roles of folate, vitamin B_12_, and iron. Annu. Rev. Nutr..

[36] Haider L.M., Schwingshackl L., Hoffmann G., Ekmekcioglu C. (2018). The effect of vegetarian diets on iron status in adults: A systematic review and meta-analysis. Crit. Rev. Food Sci. Nutr..

[37] Medawar E., Huhn S., Villringer A., Witte A.V. (2019). The effects of plant-based diets on the body and the brain: A systematic review. Transl. Psychiatry.

[38] Tong T.Y., Key T.J., Sobiecki J.G., Bradbury K.E. (2018). Anthropometric and physiologic characteristics in white and British Indian vegetarians and nonvegetarians in the UK Biobank. Am. J. Clin. Nutr..

[39] Hurrell R., Egli I. (2010). Iron bioavailability and dietary reference values. Am. J. Clin. Nutr..

[40] Clarys P., Deliens T., Huybrechts I., Deriemaeker P., Vanaelst B., de Keyzer W., Hebbelinck M., Mullie P. (2014). Comparison of nutritional quality of the vegan, vegetarian, semi-vegetarian, pesco-vegetarian and omnivorous diet. Nutrients.

[41] Lynch H., Johnston C., Wharton C. (2018). Plant-based diets: Considerations for environmental impact, protein quality, and exercise performance. Nutrients.

